# “Acute” West Nile Virus Encephalitis (Response to Krishnamoorthy et al.)

**DOI:** 10.3201/eid0905.030128

**Published:** 2003-05

**Authors:** Alexander Hindenburg, Cinnia Huang

**Affiliations:** * Winthrop University Hospital, Mineola, New York, USA

**To the Editor**: In a letter to the editor, Krishnamoorthy et al. question the diagnosis of “acute West Nile encephalitis” in our case report. We did not use the word “acute” in the paper, but the patient did in fact have an acute illness. We believe that this case report, in which West Nile virus (WNV) was isolated in cell culture, represents the best evidence for a WNV infection in a human in the United States. The diagnosis of West Nile encephalitis was based on clinical analysis (*1*); not everyone with the diagnosis undergoes an autopsy. In many instances, patients do recover. In our case, the patient had the clinical features of encephalitis consisting of unremitting fever associated with a rapid course of progressive confusion and lethargy followed by coma. In addition, increased depression of respiratory drive existed, pointing to brain stem involvement. We agree that the inflammatory changes in the brain were limited as compared to such changes in other reported cases of WNV; however, this limitation was attributable to the fact that the patient was both immunocompromized and neutropenic at the time of acute infection. Therefore, the usual inflammatory response cannot be expected. Even though the changes were limited, they were consistent with the histologic findings in previously published reports (*1*,*2*).

The second point by Krishnamoorthy et al. represents their hypothesis about a human chronic carrier state for WNV. Although a chronic carrier state is possible, the viremic period associated with arboviral infections is typically short (*3*). While one cannot rule out persistent infection with WNV, until our report attempts to recover the virus by isolation in North America in humans have been uniformly unsuccessful. Also, previous reports of successful WNV isolations by Israeli investigators in immunocompetent hosts (*4*) have been from blood specimens before seroconversion. These considerations indicate that the virus is not routinely found in the blood in substantial amounts by the time clinical symptoms consistent with WNV infection occur. We do not know, nor have we speculated, about the timing of the infection as the patient had no recollection of a mosquito bite. Tests for both immunoglobulin (Ig) G and IgM antibodies to WNV were negative in our patient. Because the patient was immunocompromised, a humoral response was not expected; therefore, this information cannot be used as evidence that the patient had an acute infection. However, observations that the patient had no manifestation of encephalitis during a previous episode of neutropenia and that she had an acute febrile illness associated with neurologic signs of encephalitis point to an acute infection. The figure, in which WNV copy numbers are correlated with leukocyte count, is not intended to pinpoint the time of infection. However, as stated in the paper, this figure did show that the virus was rapidly cleared after resolution of neutropenia.

A report by Camenga et al. (*5*) demonstrated that mice, infected with WNV develop only an inapparent infection. These mice will invariably die of fulminant encephalitis if only a single dose of cyclophosphamide is given. However, mice treated with one dose of cyclophosphamide demonstrate inflammatory changes in the brain. If a second dose of the drug is administered 5 days after infection, inflammation is completely suppressed in mice. Although mice are immunologically different from humans, this work, done almost 30 years ago, supports the argument for an acute infection in the current case report. If the patient in our study was a chronic carrier, she should have had manifestations of acute West Nile encephalitis immediately following the first course of combination chemotherapy, which was much more immunosuppressive than cyclophosphamide alone. This fact reemphasizes our major point in the article that patients who are immunocompromized and undergoing chemotherapy, which may cause neutropenia, should take extra precautions against being exposed to WNV.
